# A Secure and Fast Image Encryption Scheme Based on Double Chaotic S-Boxes

**DOI:** 10.3390/e21080790

**Published:** 2019-08-13

**Authors:** Shenli Zhu, Guojun Wang, Congxu Zhu

**Affiliations:** 1 School of Computer Science, University of South China, Hengyang 421001, China; 2 School of Computer Science, Guangzhou University, Guangzhou 510006, China; 3 School of Computer Science and Engineering, Central South University, Changsha 410083, China

**Keywords:** image encryption, compound chaotic system, S-box, image information entropy

## Abstract

In order to improve the security and efficiency of image encryption systems comprehensively, a novel chaotic S-box based image encryption scheme is proposed. Firstly, a new compound chaotic system, Sine-Tent map, is proposed to widen the chaotic range and improve the chaotic performance of 1D discrete chaotic maps. As a result, the new compound chaotic system is more suitable for cryptosystem. Secondly, an efficient and simple method for generating S-boxes is proposed, which can greatly improve the efficiency of S-box production. Thirdly, a novel double S-box based image encryption algorithm is proposed. By introducing equivalent key sequences {**r**, **t**} related with image ciphertext, the proposed cryptosystem can resist the four classical types of attacks, which is an advantage over other S-box based encryption schemes. Furthermore, it enhanced the resistance of the system to differential analysis attack by two rounds of forward and backward confusion-diffusion operation with double S-boxes. The simulation results and security analysis verify the effectiveness of the proposed scheme. The new scheme has obvious efficiency advantages, which means that it has better application potential in real-time image encryption.

## 1. Introduction

With the rapid development of network communication, image encryption has become a research hotspot in the field of image processing and information security. Since image information has the characteristics of large amounts of data, strong redundancy and high correlation between adjacent pixels, image encryption algorithms need not only high security, but also fast encryption speed. If the speed of encryption is low, the time consumed will be too long because of the large amount of image data. To encrypt multimedia information with large amounts of data, security and efficiency should be considered comprehensively [1,2,3,4,5]. Chaos-based cryptosystem just meets the need of image encryption, which leads to the research of chaos-based image encryption technology has been widely concerned by scholars. As for chaotic cryptography, a new chaotic system with better cryptographic performance deserves to be established. Some representative studies have contributed to this aspect [6,7,8,9]. How to generate key stream or encryption component with good performance is very important to the security of the image Cryptosystem [10,11,12]. How to design encryption algorithm is the core research content of the image Cryptosystem [13]. Cryptanalysis [14,15,16] is another important research direction of cryptography, which can help cryptographic designers improve the security of cryptographic algorithms.

Among many chaos-based image encryption algorithms, the permutation and diffusion (PD) pattern encryption algorithm proposed by Fridrich [17] is the most popular one. This image encryption algorithm structure consists of shuffling pixel positions and changing pixel values. The permutation (or shuffling, scrambling) process plays a role in confusing the relationship between the cipher image and plain image. The function of the diffusion process is to spread the change of one pixel value in the plain image to the whole range of the cipher image. Based on the basic confusion-diffusion architecture, researchers have proposed many novel concrete encryption strategies. In Ref. [18,19,20,21,22,23,24], authors proposed some different permutation strategies for image scrambling aiming at the confusion process. In Ref. [22,25,26,27,28,29], authors put forward some novel image diffusion algorithm. In Ref. [30,31,32,33,34,35,36], authors adopt new chaotic systems to improve the complexity and randomness of chaotic key streams. Some other cryptographic methods have also been tried by many researchers. For example, some cryptographic algorithms are based on bit-level permutation and diffusion [30], and some algorithms introduce the DNA coding mechanism [37], and some algorithms mainly use S-box to encrypt images [38,39,40]. However, some image encryption schemes exist as obvious security vulnerabilities. Thus, these image encryption schemes cannot resist some attacks, such as the chosen/known plaintext. In addition, some image encryption algorithms are inefficient, such as using bit-level image scrambling, DNA encoding mechanism, key related to plaintext Hash value [41,42], and the high-dimensional chaotic system [43,44]. Encryption algorithms with low efficiency are not suitable for some resource-constrained environments, such as mobile social network [45], sensor network communication environment [46] and searchable encryption [47]. Compared with high-dimensional continuous-time chaotic systems, low-dimensional discrete chaotic systems can generate chaotic sequences with higher efficiency. Moreover, some studies show that the complexity of discrete systems is higher than that of continuous systems [48,49,50].

Substitution-boxes (abbreviated as S-boxes) are important non-linear components in the block cipher system, which play an important role in the security of cryptosystems. Therefore, some image encryption systems based on chaos also use S-box. Majid Khan [51] employed multi-parameters chaotic systems in the construction of S-boxes that are applied to the encryption of images. The multi-parameters chaotic systems are hyper-chaotic systems. Moreover, the output trajectory points of the system need to be sampled, so the time cost of generating S-boxes in the encryption scheme is bound to be long. In addition, the S-box in the scheme is equivalent to the original key and is independent of the image content. Therefore, it is vulnerable to the chosen-plaintext attack. In order to resist the selective plaintext attack, some image encryption algorithms based on chaos introduce the mechanism of the key and plaintext association. Wang et al. [52] proposed a novel image encryption algorithm based on dynamic S-boxes constructed by chaos, in which a system up to 50 S-boxes need to be generated. It is time-consuming and unsuitable for real-time encryption. M.A. Murillo-Escobar et al. [53] proposed a color image encryption algorithm based on total plain image characteristics and 1D logistic map with optimized distribution. They have a diffusion process optimized by the modified chaotic sequence. In addition, the pseudorandom sequence for the encryption process is based on the total plain image characteristic and a 128 bits secret key, so the encryption algorithm can resist the powerful chosen-plaintext attack. Zhang et al. [54] proposed a plaintext-related image encryption algorithm based on chaos. The Zhang’s system can also fight against the chosen-plaintext attacks due to using a plaintext-related key sequence. However, in order to make the final key related to the plaintext, the process of generating the final key in the above algorithms is complex. So far, most image and video encryption algorithms based on chaos mainly rely on the empirical security analysis. However, the recent study [55] has shown that the empirical safety analysis is not enough. A encryption algorithm passing the empirical safety tests is merely a necessary condition for security, but is not a sufficient criterion.

In order to improve the security and real-time performance of the image encryption algorithm, this paper presents a simple yet security image encryption algorithm based on chaotic S-boxes. The main goal of this paper is to improve the encryption efficiency of the encryption system on the premise of ensuring a certain level of security. The main innovations of this paper are as follows: (1) A new compound chaotic system, the Sine-Tent system (STS), is proposed. The compound system has wider chaotic range and better chaotic performance than any of the original systems, so it is more suitable for cryptographic applications. (2) A simple and effective S-box construction method based on the new compound chaotic system is proposed, which can speed up the generation of S-boxes. (3) A double S-boxes based image encryption algorithm is designed. Double S-boxes can not only meet the security requirements of the system, but also make the time cost much lower than multiple S-boxes. The algorithm makes the parameters of the permutation and diffusion process interrelated and related with image ciphertext so that the encryption algorithm can resist chosen-ciphertext attack. Additionally, two rounds of forward and backward confusion-diffusion operation enhances the resistance of the system to the differential analysis attack.

The rest of this paper is organized as follows. Section 2 introduces the new Sine-Tent system (STS) model. Section 3 describes the simple and effective S-box construction method based on the Sine-Tent system. Section 4 describes the new double S-boxes based image encryption algorithm. Section 5 presents the results of experiments and analysis of the proposed scheme. Finally, some concluding remarks are given in Section 6.

## 2. The Proposed New Chaotic System

1D discrete chaotic systems have many advantages in image encryption because of their simple structures. In this section, we firstly review two 1D chaotic maps: The Sine and Tent maps. They will be used for constructing our new chaotic system. Then, a new discrete compound chaotic system is proposed to solve the problems existing in the Sine and Tent maps.

### 2.1. Sine Chaotic Map

The Sine map is one of the famous 1D chaotic maps. It is a simple dynamical system with complex chaotic behavior similar to the Logistic map. The mathematical model of the Sine map can be expressed as

**Figure 1:**
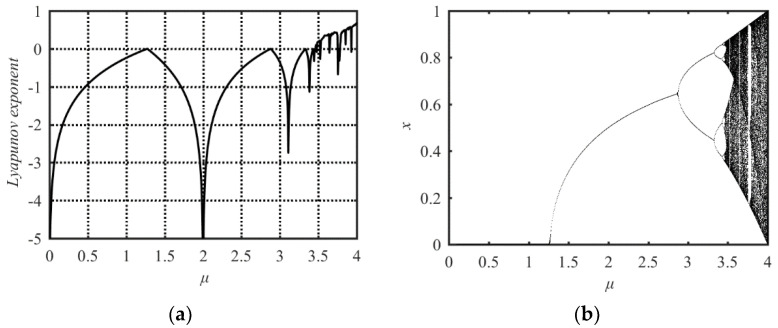
Lyapunov Exponent and bifurcation diagram of the Sine map. (**a**) Lyapunov Exponent diagram; (**b**) bifurcation diagram.

(1) x(n+1)=μ/4×sin(π×x(n))

where *μ* is the system parameter in the range of (0, 4], *x*(0) is the initial state value of the system and {*x*(*n*), *n* = 1, 2, …} is the output sequence of state values. To observe the chaotic behaviors of the Sine map, its Lyapunov Exponent and bifurcation diagram are presented in Figure 1a,b.

As is well known, for a dynamical system, a positive Lyapunov Exponent means chaotic behavior occurs in the dynamical system. So, from Figure 1a, one can see that only when the parameter *μ* ≥ 3.57 can chaotic behavior occur in the Sine map. The bifurcation diagram depicts the possible state values of the system under each parameter. Corresponding to a value of system parameter, if there are infinite state values, the system with the parameter has chaotic behavior. Corresponding to a value of system parameter, if only one or a limited number of state values output, the system with the parameter does not have chaotic behavior. In the bifurcation diagram shown in Figure 1b, the areas of *μ* with dense points shows its good chaotic behavior and the areas of *μ* with the solid line represents its non-chaotic property. There are two problems in the Sine map. First, the range of system parameters corresponding to chaotic phenomena is limited only within the range of [3.57, 4]. Even within this range, there are some parameters which make the Sine map have no chaotic behaviors. This is verified by its Lyapunov Exponent diagram and the blank zone in its bifurcation diagram. Second, when the system parameter value is less than four, the state values of the system output sequence are distributed in a narrower range than the [0, 1] interval. Only when the system parameter value is four, the state values of the system output sequence are distributed in the whole [0, 1] range. It shows the nonuniform distribution in the range of [0, 1]. These two problems reduce the application value of the Sine map.

### 2.2. Tent Chaotic Map

The name “Tent map” comes from its bifurcation diagram, which has the tent-like shape. Its mathematical model can be expressed as

(2) $$x(n+1)=\{\begin{array}{l} \mu /2\times x(n)\;\;\;x(n)<0.5\\ \mu /2\times (1-x(n))\;x(n)\geq 0.5 \end{array}$$

where *μ* is the system parameter in the range of (0, 4].

**Figure 2:**
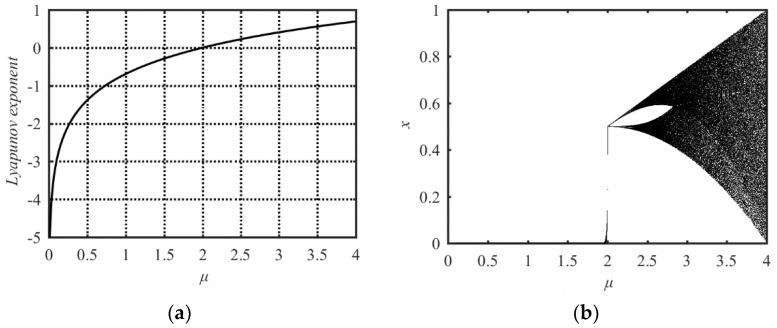
Lyapunov Exponent and bifurcation diagram of the Tent map. (**a**) Lyapunov Exponent diagram; (**b**) bifurcation diagram.

Its chaotic property is shown in the Lyapunov Exponent analysis in Figure 2a and bifurcation analysis in Figure 2b. Both analysis results indicate that its parameter value range with chaotic behavior is 2 ≤ *μ* ≤ 4. The Tent map has the same problems as the Sine map: The small parameter value range with chaotic behavior and the nonuniform distribution of the output state values.

### 2.3. The Sine-Tent System

We put forward a new compound system by combining the Sine and Tent maps and called the new system the Sine-Tent system (STS). Its mathematical model is as follows:

(3) $$x(n+1)=\{\begin{array}{l} (4-\mu )/4\times \sin(\pi \times x(n))+\mu /2\times x(n)\;\;\;x(n)<0.5\\ (4-\mu )/4\times \sin(\pi \times x(n))+\mu /2\times (1-x(n))\;x(n)\geq 0.5 \end{array}$$

where *μ* is the system parameter in the range of [0, 4]. When *μ* = 0, Equation (3) degenerates to the Sine map, while *μ* = 4, Equation (3) degenerates to the Tent map. Therefore, both the Sine map and Tent map can be regarded as special cases of the Sine-Tent system.

**Figure 3:**
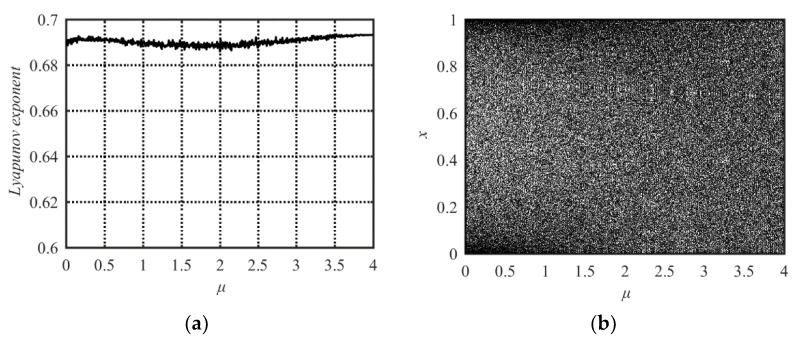
Lyapunov Exponent and bifurcation diagram of the Sine-Tent map. (**a**) Lyapunov Exponent diagram; (**b**) bifurcation diagram.

The Lyapunov Exponent and bifurcation diagram of the STS are shown in Figure 3a,b, respectively. From Figure 3 one can see that its parameter value range with chaotic behavior is *μ*∈[0, 4], which is much larger than those of the Sine or Tent maps. Its output sequences uniformly distribute within [0, 1] (see Figure 3b). Hence, the STS has better chaotic performance than the Sine and Tent maps.

The new compound system has at least three advantages compared with the Sine and Tent maps. First, the output sequences of the new compound system spread out in the entire value range between zero and one. Second, the proposed Sine-Tent system has a wider chaotic range. The Lyapunov Exponents of the Sine-Tent system is positive in the entire range of 0 ≤ *μ* ≤ 4. However, the Sine map and Tent map have positive values of Lyapunov Exponents only within much smaller ranges. Thirdly, we know that a larger Lyapunov Exponent means stronger chaotic properties. From the Lyapunov Exponent diagrams, one can see that the new system has larger Lyapunov Exponents (Lyapunov Exponents is always close to 0.7) in the whole parameter range of [0, 4], while the Sine and Tent maps have large Lyapunov Exponents only when the parameter is close to four. Therefore, the chaotic characteristic of the new system is stronger, and it always maintains the invariable excellent chaotic performance in the entire parameter range of 0 ≤ *μ* ≤ 4. These advantages guarantee that the proposed Sine-Tent system is more suitable for information security applications such as image encryption.

## 3. An Efficient New Method for Generating S-Boxes

In Ref. [56], Belazi et al. proposed a simple yet efficient S-box generating method based on the chaotic sine map, in which a prime number *p* and a one to one map from the real number interval (0, 1) to the integer set {0, 1, 2, …, 255} need to be found. In this section, we present a simpler approach for designing S-boxes using the chaotic Sine-Tent map. The new method takes advantage of the excellent chaotic characteristics of the Sine-Tent map. The detailed steps of generating S-boxes are given below.

Step 1: Set parameter *d* as an odd positive integer and *d* > 0, *d* can be used as a secret key.

Step 2: Let **T1** = 1:256, then we obtain an array **T1** which contains 256 distinct integers in the range of [1, 256].

Step 3: Based on **T1** and *d* to obtain a new array **T** by Equation (4)

(4) T(i)=mod(d×T1(i),256), i = 1, 2, …, 256

The new array **T**_1__×256_ will contain 256 distinct integers in the range of [0, 255]. As long as *d* is a finite odd integer and T1(*i*) ≠ T1(*j*) if *i* ≠ *j*, then T(*i*) ≠ T(*j*) if *i* ≠ *j*. This conclusion is true and can be proved by experimental tests.

Step 4: Set the parameters *μ*, initial state value *x*_0_ of the Sine-Tent map, and an integer *N*_0_ > 0. Iterate Sine-Tent map (*N*_0_ + 256) times to generate a chaotic sequence of length (*N*_0_ + 256). Discard the first *N*_0_ elements of the original chaotic sequence, then we can obtain a new chaotic sequence of length 256, which is represented by **X**.

Step 5: Sort the chaotic sequence **X**, then we can get a position index array **J** = {J(1), J(2), …, J(256)}, J(*i*)∈{1, 2, …, 256}. As a result of the non-periodicity of the chaotic sequence, it will inevitably lead to that J(*i*) ≠ J(*j*) as long as *i* ≠ *j*.

Step 6: Calculate the 1D array **S** as follows:

S(*i*) = T(J(*i*)), *i* = 1, 2, …, 256 (5)

Step 7: Transform the 1D array **S**_1__×256_ into a 2D matrix **S**_16__×16_, and this is the proposed S-box.

By the above method, the length of chaotic sequences to be used in constructing a 16 × 16 sized S-box is only 256. Therefore, the time cost of this method is very low. In our experiments, double S-boxes are generated by the above S-box generation algorithm. The initial condition *x*_0_, system parameter *μ* of the Sine-Tent map and the parameters {*d*, *N*_0_} for the S-box generation are set as {*x*_10_ = 0.21, *μ*_1_ = 0.399, *d*_1_ = 43, *N*_0_ = 500} and {*x*_20_ = 0.27, *μ*_2_ = 3.999, *d*_2_ = 241, *N*_0_ = 500} for S-box **S1** and **S2**, respectively. The generated double S-boxes are shown in Table 1 and Table 2, which are used in our proposed image encryption algorithm.

In the first row of Table 1, c1, c2, …, c16 denotes the column numbers of the S-box. Additionally, in the first column of Table 1, r1, r2, …, r16 denotes the row numbers of the S-box.

To determine the randomness of proposed S-box method, the statistical test suite (version 2.1.1), proposed by the National Institute of Standards and Technology (NIST) NIST-800-22 is introduced. The NIST-800-22 test results are listed in Table 3. We find that the 12 tests successfully passed. Moreover, the Random Excursions Test, Random Excursions Variant Test, and Universal Statistical Test were not applicable for the proposed S-box. This is because the sequence generated by an S-box only consists of 2048 bits. However, the Random Excursions Test and Random Excursions Variant Test require a long sequence consisting of a minimum of 1,000,000 bits, and the Universal Statistical Test also requires a long sequence consisting of a minimum of 387,840 bits.

## 4. The Proposed S-Box based Encryption Scheme

### 4.1. Cryptanalysis of an S-Box Based Encryption Algorithm

**Figure 4:**
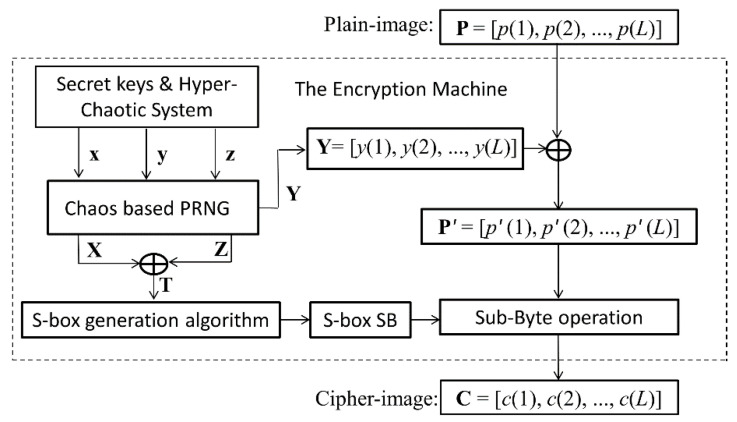
Sketch of the original encryption algorithm.

In Ref. [57], Çavuşoğlu et al. proposed an image encryption scheme by using the S-box generated with a novel hyper-chaotic system. The sketch of the encryption scheme is shown in Figure 4.

Suppose the input pixel value array of the plain image is **P** = [*p*(1), *p*(2), …, *p*(*L*)]. The output pixel value array of the cipher image is **C** = [*c*(1), *c*(2), …, *c*(*L*)]. The encryption steps can be described in detail below.

Step 1: Generate three real value chaotic sequences **x**, **y**, and **z** by using a hyper-chaotic system with given parameters and initial state values as secret keys.

Step 2: Transform the three real value sequences **x**, **y** and **z** into three integer sequences **X**, **Y** and **Z** by the chaos-based pseudo random number generator (PRNG). Each element in **X**, **Y** and **Z** is an 8-bit integer and its decimal number is in the range of [0, 255].

Step 3: The S-box, denoted as **S** = [*s*(*j*, *k*)], is created by using sequences **X**, **Z** and a novel S-box generation algorithm. Where, *s*(*j*, *k*)∈{0, 1, …, 255}, *j* = 1, 2, …, 16, *k* = 1, 2, …, 16.

Step 4: The intermediate cipher image array **P**’ = [*p’*(1), *p’*(2), …, *p’*(*L*)] is generated by using sequences **Y** = [*y*(1), *y*(2), …, *y*(*L*)] as

*p’(i)* = *y(i)* ⨁ *p(i)*, *i* = 1, 2, …, *L* (6a)

where ⨁ denotes bitwise XOR. The decryption operation corresponding to Equation (6a) can be expressed as Equation (6b):

*p (i)* = *y(i)* ⨁ *p’(i)*, *i* = 1, 2, …, *L* (6b)

Step 5: Perform sub-byte operation on **P**’ with the 16 × 16 sized S-box **S,** and obtain the cipher image array **C** = [*c*(1), *c*(2), …, *c*(*L*)].

Here, the sub-byte operation is a process in which each pixel value in the image is substituted with an element value in the S-box. The sub-byte operation can be implemented by defining a function. For example, the function sub_byte[**S**, *p*] can find a substitute to *p* from the S-box **S**. Let *q* = sub_byte[**S**, *p*], the algorithm of the function sub_byte[**S**, *p*] can be described as Algorithm 1. For example, if *p* = 55 = (0011 0111)_2_, then *j =* (0011)_2_ + 1 = 4, *k =* (0111)_2_ + 1 = 8. Consequently, *q* = sub_byte[**S**, *p*] = sub_byte[**S**, 55] = *s*(*j*, *k*) = *s*(4,8).


**Algorithm 1 The algorithm pseudo code of function *q* = sub_byte[S, *p*].**
Input:**S** = [*s*(*j*, *k*)], *p; (j* = 1, 2,…, 16, *k* = 1, 2, …, 16.)Output:*q***=** sub_byte[**S**, *p*];1:Convert *p* to a binary number (b_8_b_7_…b_2_b_1_)_2_;2:Let *j* = (b_8_b_7_b_6_b_5_)_2_ = 8 × b_8_ + 4 × b_7_ + 2 × b_6_ + 1 × b_5;_
*k* = (b_4_b_3_b_2_b_1_)_2_ = 8 × b_4_ + 4 × b_3_ + 2 × b_2_ + 1 × b_1_;3:Let *j* = *j* + 1; *k* = *k* + 1;4:Let *q* = *s*(*j*, *k*);

Therefore, Step 5 can be expressed by the following general form:

*c*(*i*) = sub_byte[**S**, *p’*(*i*)], *i* = 1, 2, …, *L* (7a)

The decryption operation corresponding to Equation (7a) can be expressed as Equation (7b):

*p’*(*i*) = sub_byte_1[**S**, *c*(*i*)], *i* = 1, 2, …, *L* (7b)

where, function sub_byte_1[·, ·] is the inverse operation of the function sub_byte[·, ·].

The above S-box based encryption algorithm has the following potential defects:

(1) The chaotic sequence **Y** and S-box is actually the equivalent of the secret keys, which are not related with the image to be encrypted.

(2) The algorithm has no diffusion effect. While one pixel is changed in the plain image, there is only one changed pixel in the cipher image.

(3) The sequence **Y** and S-box are separated in the bitwise XOR process and Sub-Byte process, and the bitwise XOR process unrelated to the Sub-Byte process.

Based on the above analysis, we find that the above encryption scheme cannot resist the chosen-plaintext attack. Suppose the target cipher image to be recovered is **C** = [*c*(1), *c*(2), …, *c*(*L*)], we can launch chosen-plaintext attack on the above encryption scheme to recover its corresponding plain image **P** = [*p*(1), *p*(2), …, *p*(*L*)]. The simplest attacking algorithm can be described as Algorithm 2.


**Algorithm 2 The simplest attacking algorithm pseudo code.**
1:*n* = 0;2:while (*n* < 256) do3: Choose the *n*-th plain image **Pn** = [*n*, *n*, …, *n*]; 4: Get its corresponding cipher image **Cn** = [*cn*(1), *cn*(2), …, *cn*(*L*)] by using the encryption machine of Figure 4;5: for *i* =1, 2, …, *L*, do
   if *c*(*i*) = = *cn*(*i*), then we can get *p*(*i*) = *n*;6: end for7: *n* = *n* + 1;8:end while

This simplest attack method with Algorithm 2 requires 256 selected plaintext images. However, a more efficient chosen-plaintext method only needs to select two plain images. For details, readers can refer to Ref. [58].

### 4.2. The Novel Double S-Boxes Based Image Encryption Algorithm

To eliminate the security defects that exist in some S-box based encryption algorithms, a novel double S-boxes based image encryption algorithm is proposed. The main innovations of the new scheme lie in the following three aspects: Firstly, the new Sine-Tent compound chaotic system is used to generate double S-boxes, which are used in the two rounds of the encryption process of the new scheme. Secondly, the first S-box is used to realize pixel confusion and substitution simultaneously. Thirdly, two rounds of the encryption process are correlated and the diffusion mechanism is introduced. The main steps of the novel double S-boxes based image encryption algorithm is described as follows:

Step 1: Input the secret parameters {*x*_10_, *μ*_1_, *d*_1_, *x*_20_, *μ*_2_, *d*_2_, *r*_0_, *t*_0_, *m*} and the plain image **PI** with the size of *M* × *N*. **PI** is reshaped to a 1D pixel array **P** = [*p*(1), *p*(2), …, *p*(*L*) ], where *L* = *M* × *N*.

Step 2: Generate the first S-box **S1** by using the new S-box generation algorithm with parameters {*x*_10_, *μ*_1_, *d*_1_}.

Step 3: Generate the second S-box **S2** by using the new S-box generation algorithm with parameters {*x*_20_, *μ*_2_, *d*_2_}.

Step 4: Perform the first round of encryption on array **P** with the first S-box **S1**, and obtain the temporary cipher image pixel array **B** = [*b*(1), *b*(2), …, *b*(*L*)] as

(8) $$\left\{ \begin{array}{l} j=\mathrm{mod}(1+m,L)+1;\\ r=r_{0};\\ b(1)=\mathrm{mod}(\mathrm{sub}\_\mathrm{byte}[S1,p(j)]+r,256). \end{array}\right.\mathrm{for}\;i\;=\;1$$

(9) $$\left\{ \begin{array}{l} j=\mathrm{mod}(i+m,L)+1;\\ r=\mathrm{mod}(b(i-1)+r,256);\\ b(i)=\mathrm{mod}(\mathrm{sub}\_\mathrm{byte}[S1,p(j)]+r+b(i-1),256). \end{array}\right.\mathrm{for}\;i\;=\;2,\;3,\;\ldots \;L$$

where, sub_byte[**S1**, *x*] denotes byte substitution for *x* using S-box **S1**. The first round of encryption is the forward confusion-diffusion operation, in which permutation and diffusion are implemented simultaneously by introducing the location index *j*.

Step 5: Perform the second round of encryption on array **B** with the second S-box **S2**, and obtain the final cipher image pixel array **C** = [*c*(1), *c*(2), …, *c*(*L*)] as

(10) $$\left\{ \begin{array}{l} t=t_{0};\\ c(L)=\mathrm{sub}\_\mathrm{byte}[S2,\mathrm{mod}(b(L)+t,256)]. \end{array}\right.\mathrm{for}\;i\;=\;L$$

(11) $$\left\{ \begin{array}{l} t=\mathrm{mod}(c(i+1)+t,256);\\ c(i)=\mathrm{sub}\_\mathrm{byte}[S2,\;\mathrm{mod}(b(i)+c(i+1)+t,256)]. \end{array}\right.\mathrm{for}\;i\;=\;L-1,\;L-2,\;\ldots ,\;1$$

where, sub_byte[S2, *x*] denotes byte substitution for *x* using S-box S2. The second round of encryption is the backward diffusion operation.

Step 6: Transform the 1D vector **C** into a 2D matrix with size of *M* × *N*, then the cipher image **CI** is obtained.

The decryption process is the inverse operation of the encryption process. To recover the plain image **P** from the cipher image **CI**, the operating steps are as follows.

Step 1: Input the secret parameters {*x*_10_, *μ*_1_, *d*_1_, *x*_20_, *μ*_2_, *d*_2_, *r*_0_, *t*_0_, *m*} and the cipher image **CI** with the size of *M* × *N*, and **CI** is reshaped to a 1D pixel array **C** = [*c*(1), *c*(2), …, *c*(*L*)], where *L* = *M* × *N*.

Step 2: Generate the first S-box S1. The operation is exactly the same as Step 2 of the encryption process.

Step 3: Generate the second S-box S2. The operation is exactly the same as Step 3 of the encryption process.

Step 4: Recover the intermediate cipher image pixel array **B** = [*b*(1), *b*(2), …, *b*(*L*)] as

(12) $$\left\{ \begin{array}{l} t=t_{0};\\ b(L)=\mathrm{mod}(sub\_byte\_1(S2,c(L))-t+256,256). \end{array}\right.\mathrm{for}\;i=\;L.$$

(13) $$\left\{ \begin{array}{l} t=\mathrm{mod}(c(i+1)+t,256)\\ b(i)=\mathrm{mod}(sub\_byte\_1(S2,c(i))-t-c(i+1)+256,256) \end{array}\right.\;\mathrm{for}\;i\;=\;L-1,\;L-2,\;\ldots ,\;1$$

where, sub_byte_1[**S2**, ·] denotes the inverse operation of sub_byte[**S2**, ·] using S-box **S2**.

Step 5: Recover the original plain image pixel array **P** = [*p*(1), *p*(2), …, *p*(*L*)] as

(14) $$\left\{ \begin{array}{l} j=\mathrm{mod}(1+m,L)+1;\\ r=r0;\\ p(j)=\mathrm{sub}\_\mathrm{byte}\_1(S1,\mathrm{mod}(b(1)-r+256,256)). \end{array}\right.\;\mathrm{for}\;i=\;1.$$

(15) $$\left\{ \begin{array}{l} j=\mathrm{mod}(i+m,L)+1;\\ r=\mathrm{mod}(b(i-1)+r,256);\\ p(j)=\mathrm{sub}\_\mathrm{byte}\_1(S1,\mathrm{mod}(b(i)-b(i-1)-r+256,256)). \end{array}\right.\;\mathrm{for}\;i\;=2,\;3,\;\ldots ,\;L$$

where, sub_byte_1[**S1**, ·] denotes the inverse operation of sub_byte[**S1**, ·] using S-box **S1**.

Step 6: Transform **P** into an *M* × *N* matrix, then the decrypted image **PI** is obtained.

## 5. Experimental Results and Security Analyses

To examine the security and efficiency of the proposed cryptosystem, we carry out some simulation experiments. All the algorithms are implemented with MATLAB R2016b run on a Microsoft Windows 7 operating system. The hardware environment is a PC with 3.3 GHz CPU, and 4 GB memory. Without losing generality, we adopted the public test images come from the USC-SIPI Image Database. Test images are 8-bit grayscale images with a size of 256 × 256, such as Lena, Baboon, Pepper. The all-black and all-white images are also used in the simulation experiments. The secret keys {*x*_10_, *μ*_1_, *d*_1_, *x*_20_, *μ*_2_, *d*_2_, *r*_0_, *t*_0_, *m*} are set as {0.21, 0.399, 43, 0.27, 3.999, 241, 98, 200, 129}.

### 5.1. Experimental Results

**Figure 5:**
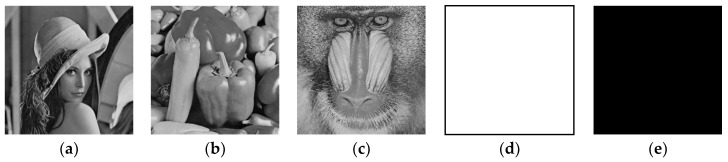
Original plain images. (**a**) The Lena plain image; (**b**) the Peppers plain image; (**c**) the Baboon plain image; (**d**) the all-white image; (**e**) the all-black image.

**Figure 6:**
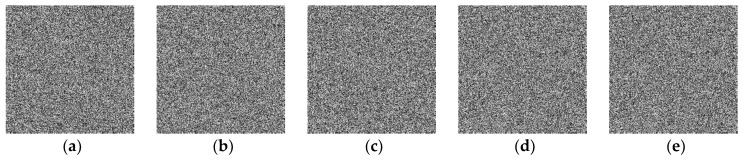
Encrypted cipher images. (**a**) The Lena cipher image; (**b**) the Peppers cipher image; (**c**) the Baboon cipher image; (**d**) the all-white cipher-image; (**e**) the all-black cipher image.

The original plain images and their corresponding cipher-images are shown in Figure 5 and Figure 6, respectively. While the decrypted images are identical to the corresponding original ones. As can be seen, the cipher-images are completely disordered and unrecognizable. Therefore, our proposed algorithm has a good encryption effect.

### 5.2. Key Space Analyses

A secure encryption scheme should have a large key space so as to resist brute-force attack. In our proposed encryption scheme, the secret keys include {*x*_10_, *μ*_1_, *d*_1_, *x*_20_, *μ*_2_, *d*_2_, *r*_0_, *t*_0_, *m*}. Among them, {*x*_10_, *μ*_1_, *x*_20_, *μ*_2_} are four double-precision real numbers, each of them can reach the accuracy of 15 decimal places. *d*_1_ and *d*_2_ are two odd integers, each of them can have 10^4^ different values. *r*_0_ and *t*_0_ are two integers, each of them has 255 different values. *m* is an integer range from 1 to *L,* where *L* = 65536. So, the key space of our proposed encryption scheme is (10^15×4+4×2^) × 255 × 255 × 65536 ≈ 2^258^, which is a key equivalent to 258 bits in length. Therefore, the key space is large enough to resist brute-force attack.

### 5.3. Statistical Analysis

#### 5.3.1. Histogram Analysis

**Figure 7:**
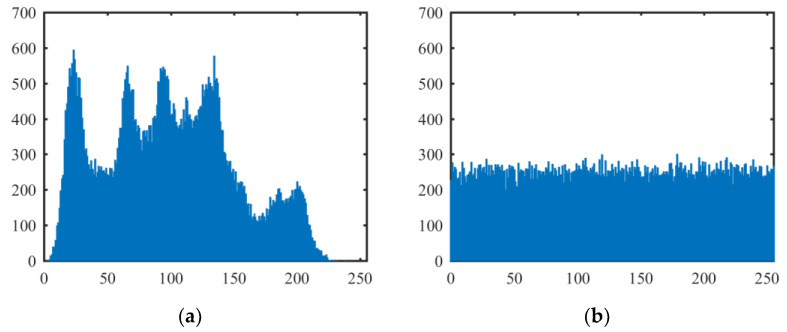

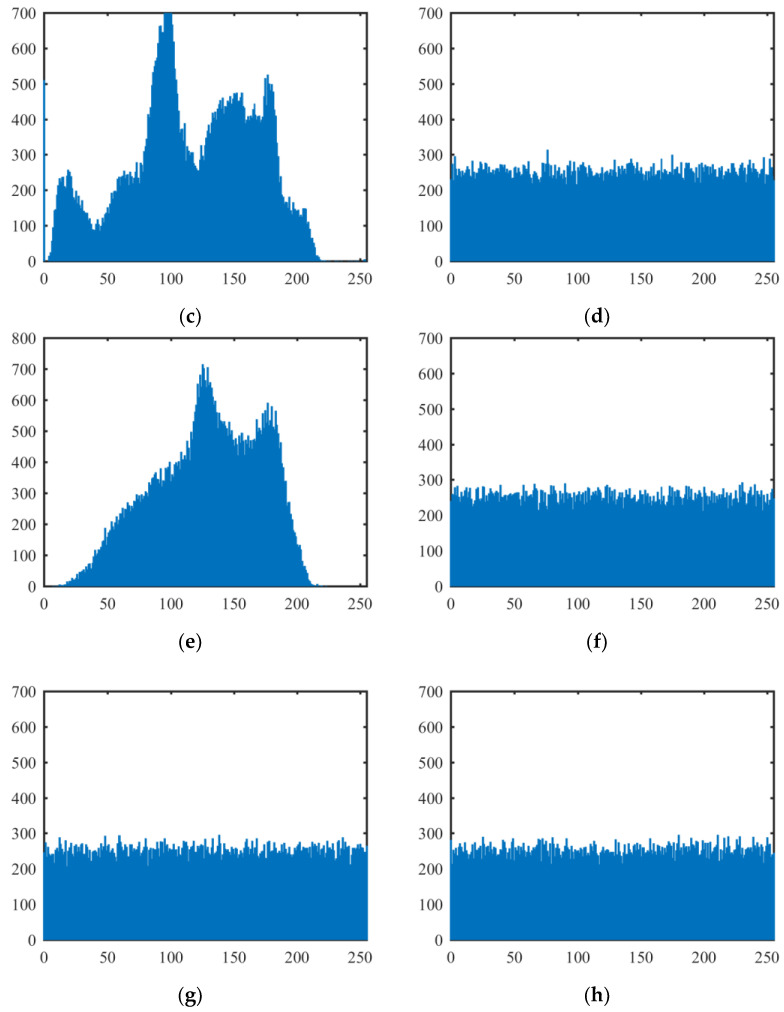
Histograms of plain images and cipher images. (**a**) The histogram of the Lena plain image; (**b**) the histogram of the Lena cipher image; (**c**) the histogram of the Peppers plain image; (**d**) the histogram of the Peppers cipher image; (**e**) the histogram of the Baboon plain image; (**f**) the histogram of the Baboon cipher image; (**g**) the histogram of the all-white cipher image; (**h**) the histogram of the all-black cipher image.

A histogram of an image demonstrates the distribution of the image pixel values, and it exposes the pixel distribution characteristics of the image. The more uniform the distribution of the pixel values, the closer the image is to the random signal image. Figure 7 shows the histograms of the above test plain images and cipher images encrypted by our proposed algorithm (the histograms of the all-white and all-black plain images are omitted). It can be seen from Figure 7 that the distributions of pixel values in plain images are clearly not uniform but in cipher images are very uniform.

The distribution characteristics of a histogram can also be described quantitatively with the variance of a histogram, which is calculated by [16]

(16) $$\mathrm{var}(Z)=\frac{1}{n^{2}}\sum_{i=1}^{n}\sum_{j=1}^{n}\frac{1}{2}{\left(z_{i}-z_{j}\right)}^{2}$$

where, *n* is the number of gray levels of an image, and *n* = 256 for 8-bit gray images. **Z** is a vector and **Z** = {*z*_1_, *z*_2_, …, *z*_n_}, *z_i_* and *z_j_* are the numbers of pixels with gray values equal to (*i* − 1) and (*j* − 1*)* respectively. The lower value of variance indicates the higher uniformity of an image. In order to detect the variance values of the above test images and their cipher images, the variances of histograms of the plain images (size of 256 × 256) and their cipher images are calculated by using Equation (16). The results are listed in Table 4. Table 4 also lists the results obtained by the algorithm in References [39] and [40]. The average variance of five cipher images obtained with our proposed algorithm is 256.7125, which is much less than that of Zhang’s algorithm [39], Wang’s algorithm [40], and Çavuşoğlu’s algorithm [57]. Thus, our proposed image encryption algorithm has better performance in resisting statistical attacks.

#### 5.3.2. Correlation Analysis

**Figure 8:**
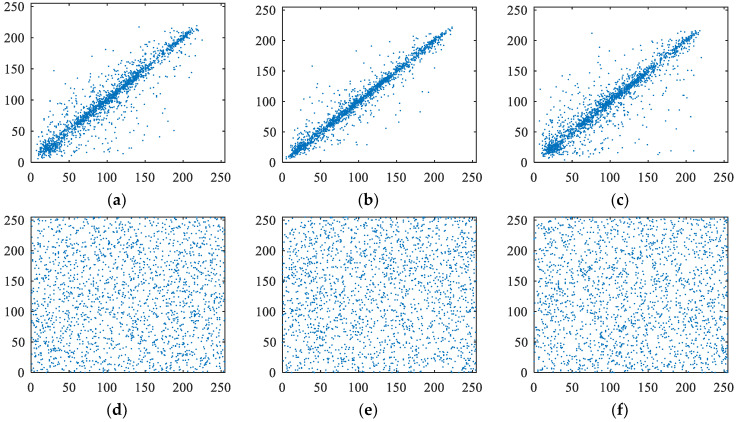
Correlation analysis of the plain and cipher Lena. (**a**) Horizontal correlation in plain image Lena; (**b**) vertical correlation in plain image Lena; (**c**) diagonal correlation in plain image Lena; (**d**) horizontal correlation in cipher image Lena; (**e**) vertical correlation in cipher image Lena; (**f**) diagonal correlation in cipher image Lena.

Natural images usually have a strong correlation with adjacent pixels. An efficient encryption algorithm should reduce the correlation in cipher images. In order to exhibit the correlation strength intuitively, we randomly selected 2000 pairs of pixel along a certain direction (horizontal or vertical or diagonal) from an image to draw the correlation distribution diagram. Figure 8 shows the correlation distribution diagrams of the Lena plain and cipher image encrypted by our encryption algorithm. The abscissa and ordinate values at any point in the graph represent the values of a pair of neighbor pixels, respectively. For plaintext images, most of the points in the graph are distributed near a straight line with an inclination of 45 degrees. That is to say, the abscissa and ordinate coordinates of most points are basically equal, indicating that the pixel values of neighboring points in plaintext images are basically equal. However, the pixel values of each group of neighbor points in ciphertext images are not equal. The results confirm that the correlation among the adjacent pixels is reduced greatly by our proposed encryption algorithm.

To illustrate quantitatively the correlation of adjacent pixels in an image, we can calculate the correlation coefficient $r_{XY}$ by using *N* pairs of an adjacent pixel. $r_{XY}$ is defined as

(17) $$r_{XY}=\mathrm{cov}\left(X,Y\right)/\sqrt{D\left(X\right)}\sqrt{D\left(Y\right)}$$

where, X = {*x*_1_, *x*_2_, …, *x*_N_} and Y = {*y*_1_, *y*_2_, …, *y*_N_}, (*x_i_*, *y_i_*) is the *i*-th pairs of the adjacent pixel gray-scale values, and

(18) $$D\left(X\right)=\frac{1}{N}{\sum_{i=1}^{N}\left(x_{i}-\bar{X}\right)}^{2},D\left(Y\right)=\frac{1}{N}{\sum_{i=1}^{N}\left(x_{i}-\bar{Y}\right)}^{2}$$

(19) $$\mathrm{cov}\left(X,Y\right)=\frac{1}{N}\sum_{i=1}^{N}\left(x_{i}-\bar{X}\right)\left(y_{i}-\bar{Y}\right)$$

(20) $$\bar{X}=\frac{1}{N}\sum_{i=1}^{N}x_{i},\bar{Y}=\frac{1}{N}\sum_{i=1}^{N}y_{i}$$

Three types of correlation coefficients of adjacent pixels in the Lena plain and cipher image are calculated, respectively. Correlation coefficients of the Lena plain images are as: 0.9567 (horizontal direction), 0.9239 (vertical direction), 0.8888 (diagonal direction), showing that correlation coefficients of adjacent pixels in the Lena plain image are very high (all close to one). Results of the Lena cipher image are listed in Table 5. From Table 5, we can see that the correlation coefficients of adjacent pixels in the Lena cipher image are very low (all close to zero). Table 5 also lists the correlation coefficients of the Lena cipher image encrypted with Zhang’s algorithm, Wang’s algorithm and Çavuşoğlu’s algorithm. The experimental results show that our proposed algorithm has the smallest absolute values of the correlation coefficient among the three algorithms, showing the best scrambling effect.

#### 5.3.3. Information Entropy Analysis

Information entropy can be used to describe the degree of randomness or uncertainty of signals. The information entropy *H*(*m*) of an image is calculated by

(21) $$H(m)=-\sum_{i=0}^{2^{n}-1}P(m_{i})\log_{2}[P(m_{i})]$$

where *P*(*m_i_*) denotes the occurrence probability of the gray level *i*, and *i* = 0, 1, 2, …*,* 2*^n^*. Here, 2*^n^* is the number of grayscale levels of an image. If each *m_i_* has the same occurrence probability in an image, then *P*(*m_i_*) = 1/2*^n^*, then the image is completely random with *H*(*m*) = *n*. For an image with 256 gray-scale levels, *n* = 8, so, the information entropy of a completely random 8-bit gray image is eight. A good encryption algorithm should make the information entropy of its cipher image close to eight. We calculated the information entropy values of several cipher images obtained by four different encryption algorithms. The results are listed in Table 6. All the images have the same size of 256 × 256. From Table 6, one can see that all the entropy values are significantly closer to eight, so the randomness is satisfactory. Among these four algorithms, our proposed algorithm has the largest average entropy value, showing the best randomness of the cipher image encrypted by our proposed algorithm.

#### 5.3.4. Sensitivity Analysis

(1) Sensitivity to plain images

A secure encryption algorithm should be sensitive to the change of the plain image so as to resist the differential attack. To measure the sensitivity of an algorithm to tiny changes in a plain image, the number of pixels changing rate (NPCR) and the unified average changing intensity (UACI) are introduced. The NPCR and UACI are calculated by Equations (22)–(24).

(22) $$\mathrm{NPCR}=\frac{1}{M\times N}\sum_{i=1}^{M}\sum_{j=1}^{N}\delta (i,j)\times 100\%$$

(23) $$\mathrm{UACI}=\frac{1}{M\times N}(\sum_{i=1}^{M}\sum_{j=1}^{N}\frac{\left|c_{1}(i,j)-c_{2}(i,j)\right|}{255})\times 100\%$$

where, *M, N* represent the number of rows and columns of an image, respectively. **C**_1_ = [*c*_1_(*i*, *j*)] and **C**_2_ = [*c*_2_(*i*, *j*)] express two encrypted images corresponding to two plain images with a tiny difference, and *δ*(*i*, *j*) is computed by

(24) $$\delta (i,j)=\{\begin{array}{c} 1,\;\;if\;c_{1}(i,j)\neq c_{2}(i,j),\\ 0,\;\;if\;c_{1}(i,j)=c_{2}(i,j). \end{array}$$

The larger the values of NPCR and UACI, the stronger the sensitivity of the algorithm to plaintext. For the best case, the ideal average value of NPCR is about 99.61%, and the ideal average value of UACI is about 33.46% [16].

To measure the sensitivity of our improved algorithm to the plain image, the original Lena gray image (size of 256 × 256) is adopted as the first plain image, and the second plain image is obtained by changing only one pixel of the first plain image. To obtain two cipher images **C**_1_ and **C**_2_ by executing the proposed encryption algorithm with the same secret keys, respectively. Then NPCR and UACI are computed with two cipher images, and the results are listed in Table 7. Table 7 also lists the results obtained by using the Zhang’s, Wang’s and Çavuşoğlu’s algorithm. The results indicate that our proposed encryption algorithm is very sensitive to the plain image, and its sensitivity is better than those of Zhang’s and Wang’s algorithm.

(2) Sensitivity to Secret Keys

A secure encryption algorithm should also be sensitive to the change of secret keys. That is to say, when secret keys change slightly, the cipher image should change dramatically. NPCR and UACI can also be used to measure the sensitivity of an encryption algorithm to secret keys. In our simulation tests, two groups of secret keys with a tiny difference are used to encrypt the same plain image Lena and two cipher images, **C**_1_ and **C**_2_, are obtained. The tiny change (to a float number is 10^−15^, or to an integer number is one) is introduced to one of the secret keys (*x*_10_, *μ*_1_, *d*_1_, *x*_20_, *μ*_2_, *d*_2_, *r*_0_, *t*_0_, *m*) while keeping all the others unchanged. The NPCR and UACI of the cipher images **C**_1_ and **C**_2_ are calculated and listed in Table 8. The experimental results indicate that our proposed algorithm is very sensitive to a slight change in any secret key.

### 5.4. Analysis of Anti-Attack Performance

#### 5.4.1. Classical Types of Attacks

According to Kerchoff’s hypothesis, it is usually assumed that the cryptanalysts or opponents know the cryptosystem, and the security entirely depends on the secret key. A secure cryptosystem should resist all kinds of attacks; otherwise, the cryptosystem is insecure. Generally speaking, there are four classical types of attacks to break a cryptosystem, and their orders from the hardest types to the easiest types are listed as follows.

(1) Ciphertext-only attack: The cryptanalyst possesses one or more ciphertexts.

(2) Known-plaintext attack: The cryptanalyst has some plaintexts and the corresponding ciphertexts.

(3) Chosen-plaintext attack: The cryptanalyst has the opportunity to use the encryption machinery, so he or she can choose some plaintext and generate ciphertext.

(4) Chosen-ciphertext attack: The cryptanalyst has the opportunity to use the cryptograph, so he or she can choose some ciphertexts and generate plaintexts.

Among the four classical attack types mentioned above, the chosen-ciphertext attack is the most powerful attack. If a cryptosystem can resist this attack, it can resist other types of attacks.

In our proposed scheme, {**S1**, **S2**, **r**, **t**} become the equivalent keys to the original keys. It is not difficult to understand the following conclusions from the encryption formulas of Equations (8)–(11). First, it is difficult for an attacker to decipher the above equivalent keys even if he or she obtains known plaintext-ciphertext pairs (*p*(*i*), *c*(*i*)). Second, the equivalent keys **r** and **t** are updated before encrypting the *i*-th pixel and they are related with the intermediate ciphertext *b*(*i*−1) or the final ciphertext *c*(*i*+1). It means that a different cipher image will yield different sequences of {**r**, **t**}. Even if the attacker cracked the key sequences of {**r**, **t**} with some specially chosen-ciphertext, the key streams of {**r**, **t**} cannot be used to decrypt the target cipher image due to the key streams of the target cipher image that are different from the cracked key streams. Moreover, it is difficult to decipher the key streams {**r**, **t**} directly by using the chosen-ciphertext attack. Therefore, the proposed scheme can well resist the chosen-ciphertext attack and can resist the four classical types of attacks.

#### 5.4.2. Analysis of Robustness against Noise and Occlusion

In order to resist the differential cryptanalysis attack brought by the opponent, a strong diffusion mechanism is introduced into the proposed encryption algorithm. As a result, the ciphertext is sensitive to the noise of the transmission channel, so the algorithm lacks robustness to noise and occlusion. However, the lack of such robustness also makes it impossible for the opponent to decipher the plaintext accurately, which can ensure that the confidentiality of the image content is protected. As for how to make the encrypted image not only resist differential attack, but also withstand a certain degree of noise, we consider introducing an error correction mechanism in channel coding and decoding. This is worthy of further study in the future.

### 5.5. Analysis of Speed

In addition to security performance, a practical cryptosystem should also have faster encryption speed. To evaluate the encryption efficiency of the proposed algorithm, the 8-bit greyscale images with a size of 256 × 256 and 512 × 512 are encrypted. And the same type of S-box based image encryption algorithms proposed by Zhang [39], Wang [40], and Çavuşoğlu [57] are also implemented on the same hardware and software platform mentioned at the beginning of Section 5. The average values of the encryption/decryption time taken by Zhang’s algorithm, Wang’s algorithm, Çavuşoğlu’s algorithm and our proposed algorithm are shown in Table 9, respectively. The experimental results show the advantages of the proposed algorithm in time efficiency.

Our proposed algorithm has an execution time that includes: Two S-boxes generated by a novel simple method using the 1D discrete chaotic map, 2*L* times of byte substitution and 2*L* times mod 256 addition operations. Zhang’s algorithm execution time include: Two S-boxes generated by an ordinary method using the 1D discrete chaotic map, 2*L* times of byte substitution, *L* times mod 256 addition operations and *L* times bitXor operations. Wang’s algorithm has an execution time that includes: Three S-boxes generated by an ordinary method using the 3D continuous-time chaotic system, *L* times of byte substitution, *L* times mod 3 addition operations and *L* times bitXor operations. Çavuşoğlu’s algorithm has an execution time that includes: One S-box generated by an ordinary method using the 3D continuous-time chaotic system, *L* times of byte substitution and *L* times bitXor operations. The mod addition operation has a less execution time than the bitXor operation, and the bitXor operation has a less execution time than the byte substitution operation. Our algorithm to generate the S-box has the least execution time among the four algorithms. As the result, the total execution time of our algorithm is the smallest one among the four algorithms.

## 6. Conclusions

In this paper, an efficient and secure image encryption scheme is presented. The main contributions of this paper are as follows: First, a new compound chaotic system, the Sine-Tent map, is proposed, which has wider chaotic range and better chaotic performance than any of the old one. And the new compound chaotic system is more suitable for cryptosystem. Second, an efficient and secure method for generating S-boxes is proposed, which has less execution time than the other ones. Third, a novel double S-boxes based image encryption algorithm is proposed. By introducing equivalent key sequences {**r**, **t**} related with image ciphertext, the proposed cryptosystem can resist the four classical types of attacks, which is an advantage over other S-box based encryption schemes. It overcomes the security defects of some old S-box based encryption algorithms. In addition, two rounds of forward and backward confusion-diffusion operation enhance the sensitivity of the algorithm. The simulation results and security analysis verify the effectiveness of the proposed scheme. The new scheme has obvious efficiency advantages, which means that it has better application potential in real-time image encryption. The proposed scheme is also suitable to color images by connecting three color channels of color image into gray image.

As for the research of the chaotic image encryption, there are two aspects worthy of further study in the future. First, we need to explore new security evaluation criteria to make up for the shortcomings of empirical security standards. Second, in order to ensure that the encryption system is not only resistant to differential cryptanalysis attacks, but also robust to noise, it may be an effective solution to introduce error-correcting codes in the process of cryptography and decoding.

## Figures and Tables

**Table 1:** The chaotic S-box **S1** generated with parameters {*x*_10_ = 0.21, *μ*_1_ = 0.399, *d*_1_ = 43, *N*_0_ = 500}.

S-box	c1	c2	c3	c4	c5	c6	c7	c8	c9	c10	c11	c12	c13	c14	c15	c16
r1	27	4	47	58	146	86	137	215	61	68	129	80	131	214	97	119
r2	168	210	253	91	219	30	112	63	52	188	73	139	55	16	158	204
r3	124	71	21	45	169	32	208	121	198	179	246	8	175	194	35	5
r4	70	3	114	42	205	89	101	159	173	127	75	235	118	243	143	141
r5	147	13	196	163	11	62	134	76	191	133	132	145	33	43	120	31
r6	17	156	245	186	25	237	88	161	0	83	87	72	116	150	255	226
r7	138	74	46	34	136	99	12	218	110	195	105	57	172	65	2	216
r8	211	184	19	20	84	242	85	98	189	22	24	185	166	109	15	217
r9	167	48	56	78	90	59	36	244	6	107	142	180	23	238	106	7
r10	28	247	199	201	40	250	206	183	223	200	29	67	128	126	10	241
r11	113	233	207	140	152	135	122	174	228	151	102	148	79	176	49	95
r12	190	103	92	39	64	1	171	220	212	51	221	130	249	170	164	230
r13	60	162	117	154	157	160	229	187	100	26	37	155	225	222	232	104
r14	181	224	53	18	108	96	66	38	248	182	178	251	165	231	202	81
r15	50	93	149	9	239	192	209	82	115	236	44	144	69	111	153	125
r16	254	41	227	213	193	14	77	197	54	123	203	177	94	252	234	240

**Table 2:** The chaotic S-box S2 generated with parameters {*x*_20_ = 0.27, *μ*_2_ = 3.999, *d*_2_ = 241, *N*_0_ = 500}.

S-box	c1	c2	c3	c4	c5	c6	c7	c8	c9	c10	c11	c12	c13	c14	c15	c16
r1	75	140	59	156	233	234	149	214	126	105	134	228	101	84	111	35
r2	113	241	53	202	17	96	93	168	172	82	78	203	159	182	249	118
r3	115	68	195	107	189	104	165	80	39	94	150	254	199	183	157	74
r4	52	210	55	200	229	48	132	163	219	201	117	146	153	43	71	230
r5	60	70	103	211	95	92	36	12	81	133	46	176	209	251	237	186
r6	98	136	20	44	178	185	177	19	137	50	21	206	65	192	129	79
r7	240	7	121	38	27	196	25	167	89	72	162	221	148	147	24	223
r8	100	47	248	164	34	29	73	69	245	1	10	191	216	26	204	18
r9	37	15	32	108	9	160	139	220	238	232	58	161	109	6	169	62
r10	45	3	0	180	114	120	246	250	33	194	198	13	158	31	66	155
r11	83	125	244	51	212	97	91	99	77	138	173	243	253	102	123	166
r12	225	208	110	40	222	87	218	197	170	184	124	131	4	112	179	255
r13	85	64	193	88	56	16	236	207	181	144	231	239	152	135	122	67
r14	151	171	42	154	142	247	28	41	14	252	224	188	54	175	217	130
r15	22	215	49	5	141	11	2	127	145	86	116	213	205	63	242	128
r16	30	226	227	106	187	23	174	190	143	8	76	61	235	119	57	90

**Table 3:** NIST-800-22 test results of the obtained S-box.

NIST-800-22 Tests	*p*-Value	Result
Frequency Test	1.00000	SUCCESS
Block Frequency Test	0.320250	SUCCESS
Cumulative Sums Test	0.536610	SUCCESS
Runs Test	0.894524	SUCCESS
Longest Run of Ones Test	1.0000	SUCCESS
Rank Test	0.481248	SUCCESS
Discrete Fourier Transform Test	0.807748	SUCCESS
Nonperiodic Template Matchings Test	0.861831	SUCCESS
Overlapping Template Matchings Test	0.282761	SUCCESS
Approximate Entropy Test	0.011732	SUCCESS
Serial Test	0.239176	SUCCESS
Linear Complexity Test	0.203697	SUCCESS
Random Excursions Test	\	TESTNOTAPPLICABLE
Random Excursions Variant Test	\	TESTNOTAPPLICABLE
Universal Statistical Test	\	TESTNOTAPPLICABLE

**Table 4:** Variances of histograms of the test images.

Images	Plain Image	Cipher Image	Cipher Image [39]	Cipher Image [40]	Cipher Image [57]
Lena	30,665.703	221.195	284.578	283.156	381.688
Peppers	36,379.133	224.234	269.727	227.898	332.898
Baboon	47,799.055	288.664	268.211	277.297	297.625
All-white image	16,711,680	293.039	544.234	41,725.063	1214.484
All-black image cipher image	16,711,680	256.430	1396.765	43,233.188	1214.484
Average	6,707,640.778	256.713	552.703	17,149.320	688.236

**Table 5:** Correlation coefficients of the Lena cipher images encrypted by different algorithms.

Algorithms	Horizontal	Vertical	Diagonal
The proposed algorithm	−0.002088	0.000312	0.001444
Zhang’s algorithm [39]	−0.000582	0.001336	−0.004690
Wang’s algorithm [40]	0.006057	0.012468	−0.006030
Çavuşoğlu’s algorithm [57]	0.001640	0.031372	−0.000626

**Table 6:** Information entropy values of several cipher images obtained by different algorithms.

Test Images	Ref. [39]	Ref. [40]	Ref. [57]	Ours
Lena cipher image	7.9969	7.9969	7.9958	7.9976
Peppers cipher image	7.9970	7.9975	7.9963	7.9975
Baboon cipher image	7.9970	7.9969	7.9967	7.9968
All-black cipher image	7.9846	7.3901	7.9871	7.9972
All-white cipher image	7.9940	7.3998	7.9871	7.9968

**Table 7:** Values of number of pixels changing rate (NPCR) and unified average changing intensity (UACI) of Lena cipher images.

Position *i*	Values	Zhang’s [39]	Wang’s [40]	Çavuşoğlu’s [57]	Ours
1	NPCR(%)	49.81	1.53 × 10^−3^	1.53 × 10^−3^	99.64
1	UACI(%)	16.86	1.14 × 10^−3^	2.75 × 10^−4^	33.55
*L*/4	NPCR(%)	74.69	1.53 × 10^−3^	1.53 × 10^−3^	99.59
*L*/4	UACI(%)	25.08	1.68 × 10^−4^	8.26 × 10^−4^	33.25
*L*/2	NPCR(%)	99.64	1.53 × 10^−3^	1.53 × 10^−3^	99.57
*L*/2	UACI(%)	33.54	6.10 × 10^−4^	4.13 × 10^−4^	33.41
*L*	NPCR(%)	49.84	1.53 × 10^−3^	1.53 × 10^−3^	99.62
*L*	UACI(%)	16.72	8.80 × 10^−4^	8.62 × 10^−4^	33.46

**Table 8:** NPCR and UACI of the proposed algorithm with a tiny difference in one of the secret keys.

Values	∆*x*_10_ = 10^−15^	∆*μ*_1_ = 10^−15^	∆*x*_20_ = 10^−15^	∆*μ*_2_ = 10^−15^	∆*d*_1_ = 1	∆*d*_2_ = 1	∆*r*_0_ = 1	∆*t*_0_ = 1	∆*m* = 1
NPCR(%)	99.63	99.62	99.56	99.62	99.61	99.58	99.63	99.61	99.61
UACI(%)	33.53	33.34	33.50	33.41	33.38	33.53	33.46	33.41	33.37

**Table 9:** The time cost tests (unit: s).

Image Size	Ref. [39]	Ref. [40]	Ref. [57]	This Paper
256 × 256	1.205	1.256	0.823	0.464
512 × 512	4.750	4.828	3.253	1.708
